# Foes or Friends? Bacteria Enriched in the Tumor Microenvironment of Colorectal Cancer

**DOI:** 10.3390/cancers12020372

**Published:** 2020-02-06

**Authors:** Siyang Xu, Wen Yin, Yuling Zhang, Qimei Lv, Yijun Yang, Jin He

**Affiliations:** State Key Laboratory of Agricultural Microbiology, College of Life Science and Technology, Huazhong Agricultural University, Wuhan 430070, Hubei, China; xusiyangxsy@gmail.com (S.X.); yinwen@mail.hzau.edu.cn (W.Y.); yulingzhang@webmail.hzau.edu.cn (Y.Z.); qimeil2017@163.com (Q.L.); yangyijuna@163.com (Y.Y.)

**Keywords:** colorectal cancer, intestinal microbiota, colorectal cancer development, tumor microenvironment, 16S rDNA sequencing

## Abstract

Colorectal cancer (CRC) is the second most commonly diagnosed cancer and the third cause of cancer death in the world, while intestinal microbiota is a community of microbes living in human intestine that can potentially impact human health in many ways. Accumulating evidence suggests that intestinal microbiota, especially that from the intestinal bacteria, play a key role in the CRC development; therefore, identification of bacteria involved in CRC development can provide new targets for the CRC diagnosis, prevention, and treatment. Over the past decade, there have been considerable advances in applying 16S rDNA sequencing data to verify associated intestinal bacteria in CRC patients; however, due to variations of individual and environment factors, these results seem to be inconsistent. In this review, we scrutinized the previous 16S rDNA sequencing data of intestinal bacteria from CRC patients, and identified twelve genera that are specifically enriched in the tumor microenvironment. We have focused on their relationship with the CRC development, and shown that some bacteria could promote CRC development, acting as foes, while others could inhibit CRC development, serving as friends, for human health. Finally, we highlighted their potential applications for the CRC diagnosis, prevention, and treatment.

## 1. Introduction

Colorectal cancer (CRC) is the collective term used for the colon, rectal and anal cancer, and is the second most commonly diagnosed cancer and the third cause of cancer death in the world. It was responsible for over 880,792 deaths and 1,849,518 new cases worldwide in 2018 [1]. As a well-known multi-factorial disease, CRC may stem from different individual genetic background, lifestyle, and environmental factors (such as diet and drugs), and from dynamic imbalance between intestinal microbiota and host immune system [2,3,4,5,6,7,8].

Intestinal microbiota is a community of microbes that live in human intestine [9]. It has been considered as an “invisible organ” of human body [10], and contains at least 150 times more genes in total than the host genome [11]. As an “invisible organ”, intestinal microbiota or their metabolites can, in fact, significantly impact human health, causing diseases such as obesity [12], diabetes [13], fatty liver disease [14], hypertension and cardiovascular disease [15], CRC [16], etc.

The composition and diversity of intestinal microbiota are influenced by both individual factors, such as age, sex, race, immune system, and environmental factors, including dietary habits and medication usage [17]. More than 1000 microbial species colonize the human intestine [18], with bacteria accounting for about 95% of the microbe population [18]. The dominant bacterial phyla in healthy individuals are *Firmicutes*, *Bacteroidetes*, and *Actinobacteria*, with *Proteobacteria* and *Verrucomicrobia* also existing in lower numbers [17]. However, in CRC patients, intestinal bacteria appear to show different profiles. In fact, there is abundant evidence to demonstrate that the composition of intestinal bacteria can potentially contribute to cancer development [7,19,20,21,22], and some intestinal bacteria involved in colorectal carcinogenesis can be described by a “driver–passenger” model [23]. The “driver” bacteria are those causing DNA damage in intestinal epithelial cell thus contributing to the initiation of CRC and formation of a tumor microenvironment that comprises cancer cells, normal cells, and the extracellular matrix they secrete [23]. These bacteria include *Bacteroides fragili* [24], *Escherichia coli* [24] and *Campylobacter jejuni* [25], which can secrete *B. fragilis* toxin (BFT), colibactin, and cytolethal distending toxin (CDT), respectively, to attack intestinal epithelial cells and cause DNA damage. The “passenger” bacteria, on the other hand, are those that are more adapted to the tumor microenvironment, occupying the niche and being able to replace the “driver” bacteria, with most of them either promoting or inhibiting the CRC development [23]. For example, *Fusobacterium nucleatum* can activate the Wnt/β-catenin pathway, stimulate cancer cell growth, and promote CRC development [26]; however, *Akkermansia muciniphila* can instead enhance the efficacy of programmed death 1 (PD-1) based immunotherapy against CRC [27]. Therefore, identifying bacteria enriched in the tumor microenvironment are important for treatment of CRC. Despite extensive research on intestinal bacteria in patients with CRC, still a large number of CRC-associated bacteria have not yet been identified. Due to the distinct individual and environment factors, these studies on CRC-associated bacteria sometimes suffer from inconsistent results. Thus, a systematic analysis of CRC-associated bacteria is required. 

In this review, via analyzing and scrutinizing the abundant 16S ribosomal DNA (rDNA) sequencing studies of intestinal bacteria in CRC patients, we conclude that twelve genera were significantly enriched in the CRC patients or tissues. We then discuss these twelve genera in detail, including their roles in the CRC development, the mechanisms for their enrichment in tumor microenvironment of CRC patients, and their application values in the CRC treatment. Our aim is to highlight new ideas for diagnosis (such as validating bacterial biomarkers), prevention, and treatment (via inhibiting carcinogenic bacteria and supplementing probiotics) of CRC.

## 2. Bacteria Correlated with CRC

We have collected and analyzed all reliable 16S rDNA sequencing studies of intestinal microbiota from CRC patients (till January 2020), but excluded those of smaller sample sizes (<6 for each group) or non-original studies. Finally, we selected 21 studies for statistical analysis on the bacterial variations in CRC patients (Table 1). We found that the abundance of 32 genera belonging to the *Bacteroidetes, Fusobacteria, Verrucomicrobia, Proteobacteria, Firmicutes*, and *Actinobacteria* phyla varied a lot (Table 2). Among them, twelve genera belong to the *Bacteroidetes*, *Fusobacteria, Verrucomicrobia*, *Proteobacteria*, and *Firmicutes* phyla are significantly enriched in the CRC patients or tissues (Table 3). Thus, we focused on the current progresses of these twelve genera in-depth below.

### 2.1. Bacteria Correlated with CRC in Bacteroidetes Phylum

#### 2.1.1. *Bacteroides*

*Bacteroides* is a Gram-negative, anaerobic, non-motile, and rod-shaped bacterium, with most of them containing no flagellum (Table 3). It normally resides in the oral cavity, upper respiratory tract, intestine and reproductive tract of human. Thomas et al. found that *Bacteroides* is abundant in the intestines of CRC patients compared to the healthy individuals [31], yet some studies found that *Bacteroides* is depleted in the intestines of CRC patients compared to the healthy individuals [34,35]. Gao et al. also reported that *Bacteroides* is abundant in CRC tissues compared to adjacent non-cancer tissues [36]; however, Carvalho et al. demonstrated that *Bacteroides* are depleted in CRC tissues compared to adjacent non-cancer tissues (Table 2) [44]. These differences may be due to the different species of *Bacteroides* existing in the intestines of CRC patients. For examples, *B. finegoldii*, *B. intestinalis*, and *B. capillosus* are significantly enriched in healthy individuals compared to the CRC patients [34], while *B. fragilis* is enriched in the intestines of CRC patients compared to healthy individuals [35].

In the *Bacteroides* genus, there is a conserved and unique genetic locus encoding polysaccharide utilization protein named commensal colonization factor (CCF) (Table 4). The expression of *ccf* is up-regulated during the colonization of *Bacteroides*, which contributes to the passage of *B. fragilis* via the colonic mucus to reside in the crypt [48]. CCF-mediated colonization of *B. fragilis* in the intestine usually requires immunoglobulin A (IgA), and CCF can regulate the level of specific capsular polysaccharides to bind IgA and thus promote *B. fragilis* colonization [49]. In addition, metalloproteinase 2 produced by *B. fragilis* can bind with the E-cadherin of host intestinal epithelial cells [50] (Table 4), suggesting that metalloproteinase 2 may contribute to colonization of *B. fragilis* in the intestine too.

**Figure 1:**
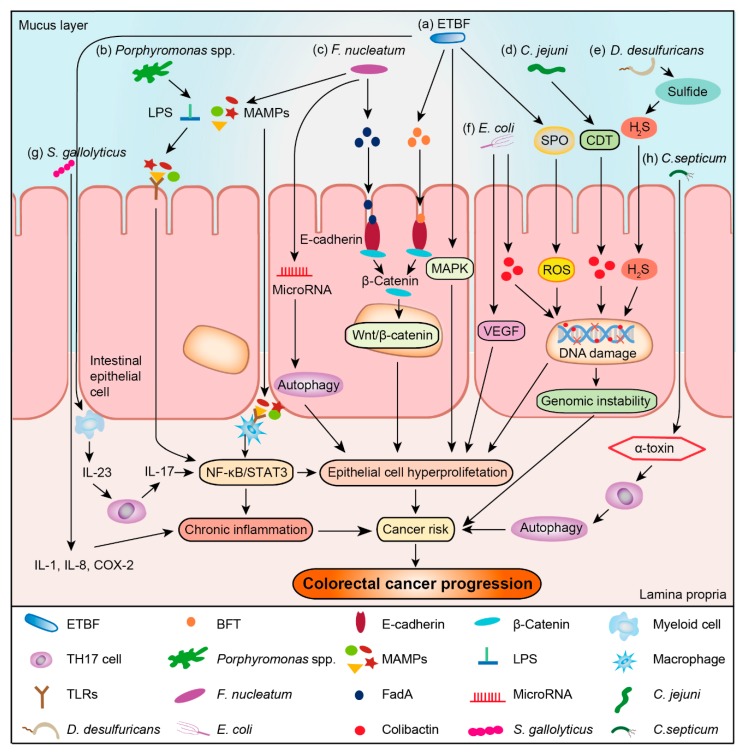
Bacteria-associated mechanisms involved in the development and progression of CRC. (**a**) Enterotoxigenic *B. fragilis* (ETBF) secretes *B. fragilis* toxin (BFT) to interact with the E-cadherin and β-catenin of host cells at adherent junctions, activate the Wnt/β-catenin signal pathway, and drive the transcription of genes related to apoptosis, cell proliferation, or transformation. Meanwhile, BFT can activate the mitogen-activated protein kinase (MAPK) signaling pathway to enhance uncontrolled proliferation of epithelial cells. Furthermore, ETBF stimulates IL-17 and IL-23 production, and induces mucosal immune response in the colonic epithelium to promote the proliferation and metabolism of colonic epithelial cells through the nuclear factor-κB (NF-κB) / signal transducer and activator of transcription 3 (STAT3) pathway. Besides, ETBF also induces spermine oxidase (SPO) to generate reactive oxygen species (ROS), thereby inducing DNA damage; (**b**) Recognition of microbe-associated molecular patterns (MAMPs), such as lipopolysaccharide (LPS) of *Porphyromonas* spp. can activate macrophages via binding its Toll-like receptors (TLRs) to activate the STAT3 or NF-κB pathway to transmit signals to the nucleus for regulating cellular growth, apoptosis, angiogenesis, migration, and cellular invasion; (**c**) *Fusobacterium* adhesin A (FadA) of *F. nucleatum* can bind E-cadherin of intestinal epithelial cells to activate β-catenin, leading to uncontrolled cell growth and acquisition of a stem cell-like phenotype. Furthermore, *F. nucleatum* is shown to modulate autophagy of intestinal epithelial cells by activating regulatory microRNAs. *F. nucleatum* also contributes to proinflammatory effects via recognition of MAMPs by TLRs, leading to the activation of the NF-κB or STAT3 pathway and accelerating CRC development; (**d**) *C. jejuni* can promote colorectal carcinogenesis through the production of cyto-lethal distending toxin (CDT), which then induces the DNA double-strand breaks, resulting in mutagenesis and chromosomal instability; these processes are also involved in cancer initiation and development; (**e**) *D. desulfuricans* can reduce sulphur or sulphur-containing compounds to hydrogen sulphide (H_2_S), which can promote oxidation and DNA damage to promote cancer development when hydrogen sulphide is present in high concentrations; (**f**) *E. coli* strains harboring the *pks* island for encoding polyketide synthases can produce genotoxin colibactin to induce DNA double-strand breaks, resulting in cancer initiation and development. *E*. *coli* can also promote vascular endothelial cell migration and increase vascular permeability by increasing the production of vascular endothelial growth factor (VEGF). All of these effects are necessary for tumor vascularization, proliferation, and migration; (**g**) *S. gallolyticus* can promote the production of inflammatory factors such as IL-1, IL-8, and cyclooxygenase-2 (COX-2), thereby accelerating CRC development through chronic inflammation; (**h**) *C. septicum* can produce α-toxin to induce apoptosis in neutrophils in the tumor microenvironment, thereby downregulating tumor immune response and accelerating CRC development.

BFT secreted by enterotoxigenic *B. fragilis* (ETBF), is a 20 kDa zinc-dependent metalloprotease toxin. It can cleave E-cadherin, reduce colonic barrier, and promote intestinal inflammation (Figure 1a) [66]. Besides, *bft* gene is more common in the intestinal mucosa of CRC patients, especially those at the terminal stage [67]. BFT can also stimulate interleukin 17 (IL-17) and IL-23 production, and mucosal immune response in the colonic epithelium, as well as promote the proliferation and metabolism of colonic epithelial cells. It can also recruit tumor-promoting myeloid cells to infiltrate and exacerbate terminal stage tumor formation [68], and activate the mitogen-activated protein kinase (MAPK) signaling, thereby promoting uncontrolled proliferation of epithelial cells (Figure 1a) [69]. Besides, ETBF can also induce spermine oxidase (SPO) to generate abundant reactive oxygen species (ROS), thereby causing DNA damage (Figure 1a) [70]. Since not all *B. fragile* can produce the toxins that accelerate CRC development, designing anti- BFT drugs may be more effective than anti-*B. fragile* bactericides for treating CRC. Meanwhile, BFT may serve as a promising biomarker for the diagnosis of CRC.

#### 2.1.2. *Porphyromonas*

*Porphyromonas* is a Gram-negative, anaerobic, non-motile and rod-shaped bacterium (Table 3). It is frequently present in the human oral cavity. The abundance of *Porphyromonas* in the intestines of CRC patients is higher than that of healthy individuals. Further, *Porphyromonas* is more abundant in CRC tissues compared to adjacent non-cancer tissues (Table 2) [32,33,35,42,45,47]. However, how *Porphyromonas* is enriched in this tumor microenvironment remains unclear.

*Porphyromonas* has been found to activate host inflammatory responses in many studies [71,72]. As shown in Figure 1b, *P. gulae* can first activate unprimed macrophages via Toll-like receptors 2 (TLR2) and Toll-like receptor 4 (TLR4), followed by inducing the effector functions of M1 macrophages via TLR2 [71]; some microbe-associated molecular patterns (MAMPs), including DNA, flagellin, lipopolysaccharide (LPS) and so on can also activate inflammatory responses, for example, the LPS of *P. gingivalis* can activate M1 and M2 macrophages by TLR2 to secrete inflammatory cytokines, such as tumor necrosis factor α (TNF-α) and IL-4 [72]. *Porphyromonas* has previously been found to be an oral pathogen, yet its role in the intestine is rarely studied. Thus, more studies are required to verify the relationship between *Porphyromonas* and CRC development.

### 2.2. Bacteria Correlated with CRC in Fusobacteria Phylum

#### 2.2.1. *Fusobacterium*

*Fusobacterium* is a Gram-negative, obligate anaerobic, non-motile and spindle-shaped bacterium (Table 3). *Fusobacterium* inhabits in human oral cavity, gastrointestinal tract, and urogenital tract. The species belong to the *Fusobacterium* genera are highly heterogeneous. Among them, some have been recognized as opportunistic pathogens involved not only in periodontitis, inflammatory bowel disease, pancreatic abscess, premature, and hepatic abscess but also in CRC and oral cancer [73,74,75]. Many studies show that *Fusobacterium* is more abundant in the intestines of CRC patients compared to the healthy individuals [32,33,36,38,42,45,46]. Comparation between CRC tissues and adjacent non-cancer tissues also reveals that *Fusobacterium* is more abundant in the CRC tissues (Table 2) [30,36,37,43,44]. These studies indicate that there is a close relationship between *Fusobacterium* and CRC development.

**Figure 2:**
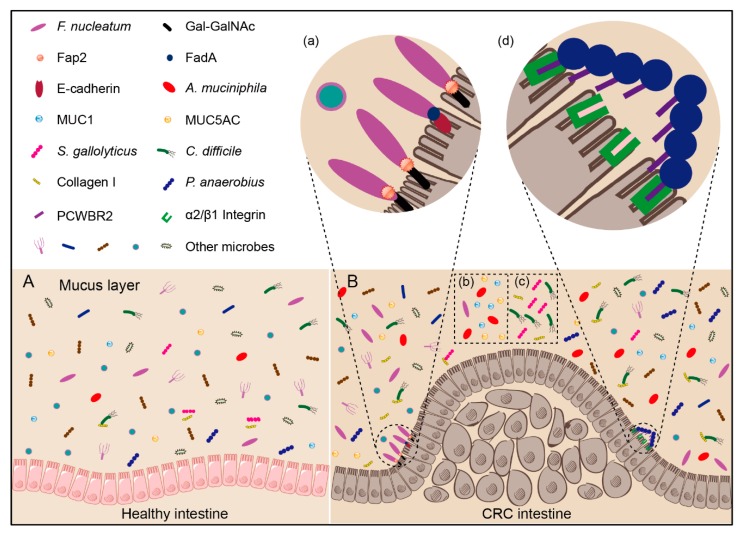
Potential mechanism of bacterial enrichment in CRC tissues. (**A**) Healthy intestine. (**B**) CRC intestine: (**a**) Gal-GalNAc is overexpressed in CRC cells, onto which the Fap2 of *F. nucleatum* can directly bind, resulting in specific *F. nucleatum* accumulation in CRC tissues; on the other hand, FadA can help *F. nucleatum* adhere to E-cadherin of intestinal epithelial cells; (**b**) MUC1 and MUC5AC are increased in the intestine of colorectal cancer patients. Due to the increasing available substrates, the abundance of *A. muciniphila* in the intestine of colorectal cancer patients is thus increased; (**c**) Collagen I is increased in the intestine of colorectal cancer patients. *S. gallolyticus* and *C. difficile* can combine with collagens I, resulting in increased abundance in the intestine of colorectal cancer patients; (**d**) The surface protein PCWBR2 of *P. anaerobius* can directly interact with the colorectal epithelial cell via α2/β1 integrin, which is frequently overexpressed in CRC tissues.

As an oral bacterium, how can *Fusobacterium* colonize colorectal tissues? Interestingly, adhesion protein Fap2 of *F. nucleatum* has been found to bind to the D-galactose-β(1–3)-N-acetyl-D-galactosamine (Gal-GalNAc), which is over-represented in CRC cells (Figure 2a and Table 4) [51]. Further, *Fusobacterium* adhesin A (FadA) can help *F. nucleatum* adhere to E-cadherin of intestinal epithelial cells (Figure 2a and Table 4) [26]. *F. nucleatum* can thus selectively colonize in CRC tissues with the assistance of Fap2 and FadA. When injected into the tail veins of precancerous and malignant CRC mouse models, *F. nucleatum* is also found to colonize in the CRC tissues [51], suggesting that *F. nucleatum* uses a hematogenous route to reach CRC tissues from the oral cavity.

In the *Apc^Min/+^* mouse model of CRC, *F. nucleatum* can increase tumor multiplicity, activate nuclear factor kappa-B (NF-κB) pathway, and drive myeloid cell infiltration into tumors to generate a pro-inflammatory environment that promote CRC development (Figure 1c) [20]. There is also a report that *F. nucleatum* can increase the proliferation of CRC cells and tumor development via TLR4 signaling activation. Indeed, *F. nucleatum* can target the TLR4 and myeloid differential protein-88 (MyD88) innate immune signaling with specific microRNAs to activate the autophagy pathway, leading to different CRC chemotherapeutic response to promote CRC resistance to chemotherapy (Figure 1c) [76]. The virulence factor FadA can promote E-cadherin-mediated tumor growth and induce host to produce proinflammatory cytokines, with the *fadA* gene levels in the colon tissues from patients with adenomas and adenocarcinomas being >10–100 folds higher than healthy individuals (Figure 1c) [26]. Fap2 protein of *F. nucleatum* can also directly interact with the T-cell immunoreceptor with Ig and ITIM domains (TIGIT) protein, inhibiting of natural killer cell cytotoxicity [77]. 

These studies indicate that *Fusobacterium* can not only serve as a CRC biomarker for the diagnosis and prognostic assessment, but also as a potential therapeutic target for CRC. 

#### 2.2.2. *Leptotrichia*

*Leptotrichia* is a Gram-negative, anaerobic, non-motile and straight or slightly curved bacterium (Table 3). It is found mostly in the oral cavity and some other parts of the human body, such as human gastrointestinal tract, periurethral region, and the genitalia of women [78]. The abundance of *Leptotrichia* is lower in CRC patients compared to healthy individuals [27]. After comparing the CRC tissues with adjacent non-cancer tissues, de Carvalho et al. showed that the abundance of *Leptotrichia* is decreased in the CRC tissues [44]. However, Xu and Jiang instead found that *Leptotrichia* is more abundant in the CRC patients (Table 2) [46]. These different results may be due to: (1) The sample size is insufficient to reach a distinct conclusion; and (2) The right colon cancer and left colon cancer may exhibit some differences. For example, *Leptotrichia* are more abundant in the left colon cancer patients compared to the right colon cancer patients [79], but this study didn’t seem to contain control samples from healthy individuals. More studies are required to verify the relationship between *Leptotrichia* and CRC development. 

### 2.3. Bacteria Correlated with CRC in Verrucomicrobia Phylum

#### *Akkermansia* 

*Akkermansia* is a Gram-negative, obligate anaerobic, non-motile and elliptical-shaped bacterium (Table 3). It is also an intestinal symbiotic bacterium colonizing in the mucosal layer of human intestine. In fact, *A. muciniphila* can use mucin as its sole sources of carbon, nitrogen, and energy, and is thus considered to be a promising candidate as probiotics. It may keep host-intestinal microbiota balance by converting mucin into beneficial by-products [80]. In fact, clinical studies reveal that the abundance of *Akkermansia* is generally decreased in individuals with metabolic impairments [81]. To date, no evidence can prove that *A. muciniphila* alone can cause pathogenicity. In fact, *A. muciniphila* can alleviate a range of diseases, including type I diabetes [82], alcoholic liver [83], progeroid [84], cancer [27], and obesity [85].

Many studies show that *A. muciniphila* has a higher relative abundance in CRC patients compared to healthy individuals (Table 2) [32,34,37]. For example, Byrd and Bresalier found that the expression of mucin 1 (MUC1) is increased in colon cancers and that of mucins 5AC (MUC5AC) is frequently present in colorectal adenomas and colon cancers [52] (Table 4), indicating that the enrichment of *A. muciniphila* in tumor microenvironment may result from the increased substrate concentration (Figure 2b). However, there is no report yet that *A. muciniphila* can promote the development of CRC.

A recent study also revealed that *A. muciniphila* is especially enriched in cancer patients responding to PD-1 treatment compared to non-responders [27]. In mouse models, after treatment of fecal microbiota transplantation (FMT) with non-responders’ feces and along with oral supplementation with *A. muciniphila*, the mouse can restore efficacy of PD-1 blockade [27]. Based on its enrichment in intestines of CRC patients and the probiotic effect, *A. muciniphila* has thus the potential to serve as an anticancer probiotic.

### 2.4. Bacteria Correlated with CRC in Proteobacteria Phylum

#### 2.4.1. *Campylobacter*

*Campylobacter* is a Gram-negative, aerobic or anaerobic, motile and curved-shaped bacterium (Table 3). It is typically found in the human intestine and invades the lining of the human small intestine. Many studies revealed that *Campylobacter* is enriched in the CRC tissues compared to adjacent non-cancer tissues [37,44]. Meanwhile, the abundance of *Campylobacter* in the intestines of CRC patients is higher than that of healthy individuals (Table 2) [33,38,46]. 

The colonization of *C. jejuni* in the intestine is mediated by the binding of *Campylobacter* adhesin to fibronectin (CadF), fibronectin-like protein (FlpA), and permease PEB1 with their corresponding targets (Table 4). Both CadF and FlpA can bind to fibronectin, which is the main ingredient of the extracellular matrix of intestinal epithelial cells, leading to the colonization of *C. jejuni* in the intestine [53,54]. The colonization mechanism mediated by PEB1 is unclear, but the colonization capacity of PEB1-deleted strains is found to reduce by 50–100 folds [55]. In addition, FliD, a terminal cap protein of flagella in *C. jejuni*, can interact with the heparan sulfate glycosaminoglycan receptors on the intestinal epithelial cell surface [56], thus facilitating the *C. jejuni* colonization in intestine (Table 4).

Campylobacter is reported to be associated with development of inflammatory bowel disease, which can increase the risk for CRC [86]. Further, *C. jejuni* can promote CRC development through the action of CDT in Germ-free (GF) *Apc^Min/+^*mice (Figure 1d) [87]. In short, *C. jejuni* can accelerate the development of CRC, but the mechanisms for *Campylobacter* enrichment in CRC tissues remain unknown. Further researches are needed in this area to reach a conclusion.

#### 2.4.2. *Desulfovibrio*

*Desulfovibrio* is a Gram-negative, obligate anaerobic, motile and curved- or spiral rod-shaped bacterium (Table 3). It can exist under different habitats, including human intestine. *Desulfovibrio* is one of the sulphate-reducing bacteria that serve as a terminal oxidant to anaerobically degrade organic matter entering the gastrointestinal tract [88]. *Desulfovibrio* is more abundant in the intestines of CRC patients than in healthy individuals (Table 2) [31,33,38].

*Desulfovibrio* can extensively use various substrates, including hydrogen, alcohols, short-chain fatty acids, other organic acids, and amino acids, to reduce sulphur or sulphur-containing compounds to hydrogen sulphide (H_2_S) [88]. High concentrations of H_2_S can inhibit cytochrome C oxidase (complex IV), subsequently disrupting mitochondrial electron transport. Additionally, H_2_S can also promote oxidation and DNA damage to promote cancer development (Figure 1e) [89]. These studies may explain the role of *Desulfovibrio* in cancer development. Besides, the LPS of *D. desulfuricans* is capable of modulating transcriptional activity of NF-κB, p65, and IκBα encoding genes in colon cancer cells [90]. Although there is no direct evidence that *Desulfovibrio* can cause CRC, *Desulfovibrio* is possibly playing a role in the development of CRC.

#### 2.4.3. *Escherichia/Shigella*

*Escherichia/Shigella* are Gram-negative, facultative anaerobic and rod-shaped bacteria, with *Escherichia* being motile while *Shigella* being non-motile (Table 3). Both can colonize in the human intestines, and are mostly harmless, but certain strains can be very contagious, resulting in painful abdominal cramps, diarrhea, and fever. *Escherichia/Shigella* show significant different abundance between CRC patients and healthy individuals. Some studies indicate that CRC patients have higher abundance of *Escherichia/Shigella* [33,35,36,45], whereas other studies instead reveal that those of *Escherichia/Shigella* are decreased in the rectal cancer patients compared to healthy individuals (Table 2) [31]. 

Although *E. coli* normally inhabits in the human intestine. Prorok-Hamon et al. identified an *afa*-1 operator in the colonic mucosal *E. coli*, which encodes afimbrial adhesin to adhere and invade intestinal epithelial cells (Table 4) [58]. Meanwhile, they confirmed that Afa-1 can up-regulate VEGF expression in epithelial cells, promoting angiogenesis and CRC development (Figure 1f) [58]. In fact, bacterial adhesion protein intimin, which is encoded by the *eae* gene, can also help *E. coli* attach closely to the intestinal mucosa [57] (Table 4). In addition, phylogenetic analysis shows that *E. coli* comprises four main phylogenetic groups (A, B1, B2, and D), and most strains of group A and D are highly adherent to the intestinal epithelial cells; on the other hand, strain of group B2 instead displays low level of adhesion to intestinal epithelial cells [91].

*E. coli* strains of group B2 also harbor a genomic island called “*pks*”, which encodes a hybrid polyketide-peptide genotoxin of colibactin. Deletion of this *pks* island from *E. coli* NC101 is found to cause decreasing tumor multiplicity and invasion in the AOM/IL10^-/-^ mice without altering intestinal inflammation [92]. Colibactin toxin can attack host DNA directly, via introducing double-stranded DNA breaks that give rise to genomic instability, leading to increased mutation frequency and risk of CRC (Figure 1f) [93]. In addition, the encoding gene of colibactin is over-represented in *E. coli* isolated from CRC patients [94]. These data suggest that colibactin is a carcinogenic toxin. Besides, there are also other toxins produced by *E. coli*, such as CDT that can induce a remarkable cell distension, leading eventually to cell death [95]; cytotoxic necrotizing factor (Cnf) that induce dysfunctions in transformed epithelial cells [96]; and cycle inhibiting factor (Cif) that induces the formation of stress fibers and blocks cell cycle G2/M transition [97]. Because not all *E. coli* produce toxins, the carcinogenicity of *E. coli* may depend specifically on the levels of toxin gene rather than the total *E. coli* abundance. The toxins genes of *E. coli* are thus more suitable as CRC biomarker than intact *E. coli.*

### 2.5. Bacteria Correlated with CRC in Firmicutes Phylum

#### 2.5.1. *Streptococcus*

*Streptococcus* is a Gram-positive, facultative anaerobic or obligate anaerobic, non-motile and round- or ovoid- shaped bacterium (Table 3). It is widely present in nasopharynx of healthy human, with most of them being non-pathogenic. Many studies have demonstrated that *Streptococcus* is more abundant in the intestines of CRC patients than healthy individuals [33,35,46,47]. Besides, the abundance of *Streptococcus* in the CRC tissues is higher than that of adjacent non-cancer tissues (Table 2) [36]. These evidences suggest that *Streptococcus* is likely involved in the development of CRC. 

*S. gallolyticus* subsp. *gallolyticus*, which is also known as *Streptococcus bovis* biotype I, bears a strong association between invasive infections with *S. gallolyticus* and CRC by meta-analysis in clinical practice [98]. Through investigating the relationship between the subtypes of *S. bovis* and CRC, the authors found that there are 71% association between *S. gallolyticus* and CRC and 17% association between *S. bovis* biotype II bacteraemia and CRC [99]. These evidences suggest that the relationship between *Streptococcus* and CRC is mainly caused by *S. gallolyticus*. 

The molecular mechanism of *S. gallolyticus* colonization on CRC tissues has also been revealed. By assessing the ability of 17 species of *S. bovis* group to adhere to components of the extracellular matrix in *vitro*, Sillanpaa et al. found that *S. gallolyticus* exhibits stronger binding ability to collagens I and IV than *S. bovis* [59] (Table 4). Importantly, collagen IV is the main components of basement membrane of colon mucosa in the lamina propria mucosae [100] and is highly expressed in the desmoplasia of CRC liver metastases patients [60], but type IV collagenases (MMP-2 and MMP-9) are significantly up-regulated in the basement membrane during CRC development, leading to collagen IV degradation [101]. Collagen I is increased in serum and tissues of CRC patients, which is dynamically changed during stages I-IV of CRC, with the maximum expression in stage II (Figure 2c) [61]. *S. gallolyticus* produces a bacteriocin (gallocin) to kill *Enterococcus* and help it gain a more favorable environment. Additionally, the bacteriocin can be enhanced by bile acids, which is significantly increased in CRC patient [102]. These studies seem to explain the enrichment of *S. gallolyticus* in CRC tissues.

*S. gallolyticus* can accelerate the development of CRC, and Abdulamir et al. found that *S. gallolyticus* may aggravate the tumor microenvironment through inflammatory factors such as cyclooxygenase-2 (COX-2), IL-1 and IL-8, followed by accelerated CRC development (Figure 1g) [103]. After co-incubating CRC cells with *S. gallolyticus*, the authors found that CRC cells exhibit increasing levels of c-Myc, β-catenin, and proliferating cell nuclear antigen to promote colon tumor development, leading to larger tumors and dysplasia grade in CRC mouse model [104]. 

These results demonstrate that *S. gallolyticus* can accumulate in the tumor microenvironment and accelerate the development of CRC. Therefore, as a carcinogenic bacterium, it may also serve as a new target for CRC treatment.

#### 2.5.2. *Clostridium*

*Clostridium* is a Gram-positive, anaerobic, motile and rod-shaped bacterium (Table 3). They produce spores and are highly resistant to the outside environment. It mainly resides in the human intestine, and some studies indicate that the abundance of *Clostridium* in the intestines of CRC patients is higher than that of healthy individuals [31,32,46]. Further, *Clostridium* population is significant enhanced in the CRC tissues compared to the adjacent non-cancer tissues (Table 2) [44]. In a test group including 781 subjects, the authors found that the abundance of *C. symbiosum* gradually increases from the colorectal adenoma (CRA), early CRC, to advanced CRC [105]. The abundance of *C. symbiosum* and the fecal immunochemical test have also been used in combination to diagnose early CRC [105]. 

Several mechanisms have been proposed for *Clostridium* colonization (Table 4): (1) A cell biology experiment demonstrated that the adhesin Cwp66 protein in *C. difficile* can bind to Vero cells [63]; (2) The surface-layer protein A (SlpA), which is the most abundant protein of the S-layer, can significantly affect the adhering of *C. difficile* to intestinal epithelial cells, and *C. difficile* with different subtypes of SlpA is found to exhibit different adhesion abilities [62]; (3) Fbp68, which is located on the surface of *C. difficile*, can bind to fibronectin to play an important role for the *C. difficile* adherence to initiate infection [64]; (4) Collagen binding protein A (CbpA), which exhibits a high affinity to both collagen I and collagen V, is also found to contribute to the colonization of *C. difficile* on the intestine [106]. Because the abundance of collagens I is increased in serum and tissues of CRC patients, CbpA may be one of the key factors for the accumulation of *C. difficile* in CRC tissues (Figure 2c).

*Clostridium* comprises not only pathogenic bacteria that promote CRC development but also probiotics that inhibit CRC development. Indeed, *C. septicum* produces α-toxin that induces apoptosis in neutrophils in the tumor microenvironment, thereby down-regulating tumor immune response and accelerating CRC development (Figure 1h) [107]. Meanwhile, *C. difficile* secretes two toxins, TcdA and TcdB, which inactivate Rho GTPase to interrupt cell-to-cell connection and increase permeability of the intestinal barrier. Moreover, TcdA and TcdB can stimulate epithelial cells and immune cells to secrete cytokines such as TNF, IL-1, IL-5, and IL-8 to activate inflammatory responses [108]. However, *C. butyricum*, a butyrate-producing probiotic, can relieve intestinal inflammation, improve immune homeostasis, and inhibit CRC development in mice [109]. In addition, *C. butyricum* can improve the intestinal microbiota composition and suppress the Wnt/β-catenin signaling pathway, significantly inhibiting the development of CRC in *Apc*^Min/+^mice [110].

These results indicate that some of *Clostridium* can promote CRC development, while others instead inhibit CRC development. How to reduce the pathogenic effect of *Clostridium* and increase the beneficial effect of *Clostridium* in the intestines of CRC patients will be a new strategy for treating CRC.

#### 2.5.3. *Parvimonas*

*Parvimonas* is a Gram-positive, obligate anaerobic, non-motile and round-shaped bacterium (Table 3), which is frequently found in the human oral cavity. It is enriched in CRC and oral cancer patients, with its abundance in the intestines of CRC patients being higher than in healthy individuals [33,45,46]. In the CRC patients, *Parvimonas* exhibits higher abundance in CRC tissues compared to adjacent non-cancer tissues (Table 2) [30,44]. 

So far, there are few reports referring to the correlation between *Parvimonas* and CRC, and there is also no animal or cell biology experiment to prove that *Parvimonas* can cause cancer or accelerate the development of cancer. The mechanism by which *Parvimonas* is enriched in CRC tissues remains unclear and need to be further studied.

#### 2.5.4. *Peptostreptococcus*

*Peptostreptococcus* is a Gram-positive, obligate anaerobic, nonmotile and spherical- or oval- shaped bacterium (Table 3), which is found in human oral cavity, upper respiratory tract, intestine, and female reproductive tract. *Peptostreptococcus* is considered to be a carcinogenic bacterium that promotes the development of CRC, and many studies have found that the abundance of *Peptostreptococcus* in the intestines of CRC patients is higher than that of healthy individuals [32,33,35,36,42,46]. In CRC patients, *Peptostreptococcus* also exhibits higher abundance in CRC tissues compared to adjacent non-cancer tissues (Table 2) [30,44].

Long et al. first recognized the molecular mechanism of *P. anaerobius* enrichment in CRC tissues. They identified a surface protein of *P. anaerobius*, PCWBR2, which can directly interact with the colorectal epithelial cell via α2/β1 integrin that is frequently overexpressed in CRC tissues (Figure 2d, Table 4). This data confirms that *P. anaerobius* is selectively enriched in CRC tissues. Further, interaction between α2/β1 integrin and PCWBR2 can activate the PI3K-Akt pathway in CRC cells, leading to the activation of NF-κB and enhanced cell proliferation that subsequently accelerates the development of CRC [65]. Besides, *P. anaerobius* can enhance intestinal dysplasia in mice treated with azoxymethane, because *P. anaerobius* interacts with both TLR2 and TLR4, and such reactions increase the level of intracellular reactive oxidative species, to promote the cholesterol synthesis and cell proliferation, finally leading to accelerated cancer development [111]. These results show that *P. anaerobius* can be enriched in the tumor microenvironment and participate in the development of CRC.

## 3. Conclusions and Prospects

If the battle between human and CRC is a “prolonged war”, the tumor microenvironment would be the front line of the battlefield. Are the bacteria that enriched in tumor microenvironment foes or friends? Identification and clarification of the relationship between these bacteria and CRC development are extremely important.

In this review, we focused on the analyses of twelve genera that are enriched in the tumor microenvironment of CRC patients and explored their relationship with CRC development. These bacteria can be divided into three groups: (1) Direct carcinogenic bacteria, like *F. nucleatum*, *S. gallolyticus*, *C. difficile*, and *P. anaerobius*. Scientists have proposed the potential mechanism of their enrichment in CRC microenvironment, and found that these bacteria can directly participate CRC development; (2) Indirect carcinogenic bacteria, like enterotoxigenic *B. fragile* and *E. coli*. They act indirectly to impact CRC pathogenesis via secondary metabolites, or induction of immune changes in the tumor microenvironment. *B. fragile* and *E. coli* are such bacteria that are not specifically enriched in the tumor microenvironment, but the toxins they produced can promote the development of CRC; (3) Anticancer probiotics, like *A. muciniphila*. They do not promote development of CRC and are beneficial to human health. 

We conclude that there are three mechanisms for bacteria to affect CRC development: (1) They stimulate the immune system and thereby trigger chronic inflammation. Processes in chronic inflammation might cause or facilitate epithelial cell hyper-proliferation, oncogene activation, and angiogenesis; (2) They directly or indirectly damage host DNA. Occasionally, DNA damage surpasses the host cell repair capacity, and such incomplete DNA repair would result in mutagenesis and genomic instability, leading to CRC initiation and development; (3) They affect cell proliferation and cellular apoptosis through activation of NF-κB or β-catenin signaling. This could promote tumor development by regulating the expression of anti-apoptotic, cell cycle or pro-inflammatory proteins. Bacteria could bind E-cadherin on the colonic epithelial cells and triggered β-catenin activation, resulting in dysregulated cell growth to acquire stem cell–like qualities. 

The bacteria enriched in tumor microenvironment have many known or potential application prospects for the CRC diagnosis, prevention, and treatment, including: (1) For strains enriched in the intestines of CRC patients, they can be regarded as biomarkers, and it is possible to develop a diagnostic method for CRC, such as qPCR and other cheap and fast methods to detect the abundance of these bacteria in the patients’ feces to screen for high-risk CRC population; (2) For carcinogenic bacteria enriched in CRC, drugs against them can be developed to reduce its abundance in CRC patients, thus inhibiting the CRC development; (3) For probiotics that are colonized in the tumor microenvironment, one can increase their abundance in the intestines with oral supplements to improve CRC patients’ health. These can be enclosed and supplied in a specific CRC drug delivery vehicle to target the tumor site of CRC, release cancer treatment drugs, and exert their probiotic effect.

In the future, more research on the CRC and intestinal bacteria, standardized analysis, and CRC mouse models are required to better understand how these bacteria can be used to efficiently prevent or treat CRC. If we can clearly understand the relationship between these bacteria and CRC development, we can also use bacteriophages, targeted antibiotics or even develop new vaccines to fight against these bacteria to develop new strategies for the CRC treatment.

## Figures and Tables

**Table 1:** Source of data for this review.

Area	Group /Number	16S rDNA Region	Group /Specimens Type	Intestinal Microbiota Alter in Tumor Microenvironment of CRC Patients	Ref.
China	CRC/9; Control/14	V6	CRC/T Control/T	*Devosia* ↑ *Eubacterium* ↓	[28]
France	CRC/58 Control/9	V3-V4	CRC/Pt Control/T	Right-side tumors: *Fusobacterium* ↑ *Bacteroides fragilis* ↑ Left-side tumors: *Parvimonas micra* ↑	[29]
China	CRC/65	V4	CRC/Pt	*Fusobacterium* ↑ *Dermabacter* ↑ *Mucispirillum* ↓	[30]
Brazil	CRC/18 Control/18	V4–V5	CRC/T Control/T	*Parcubacteria* ↑ *Planctomycetes* ↓	[31]
Morocco	CRC/11 Control/12	V1–V2	CRC/S Control/S	*Fusobacterium* ↑ *Clostridia* ↑ *Bacteroidi*a ↓ *Slackia* ↓	[32]
Ireland	CRC/59 Control/56 Polyps/21	V3-V4	CRC/Pt, S; Control/T, S; Polyps/T, S.	*Bacteroidetes* Cluster 2 ↑ *Firmicutes* Cluster 2 ↑ *Pathogen* Cluster ↑ *Prevotella* Cluster ↑ *Bacteroidetes* Cluster 1 ↓ *Firmicutes* Cluster 1 ↓	[33]
USA	CRC/74 Control/94	V3-V4	CRC/S Control/S	*Fusobacterium* ↑ *Porphyromonas* ↑ *Clostridia* ↓	[3]
USA	CRC/10 Control/11	V4	CRC/S Control/S	*Akkermansia muciniphila* ↑ *Citrobacter farmeri* ↑ Butyrate-producing species ↓	[34]
China	CRC/46 Control/56	V3	CRC/S Control/S	*Bacteroides fragilis* ↑ *Entcreptococcus* ↑ *Bacteroides vulgatus* ↓ *Bacteroides uniformis* ↓ *Roseburia* ↓ Butyrate-producing bacteria ↓	[35]
China	CRC/31 Control/30	V3	CRC/Pt Control/ T	*Lactococcus*↑ *Fusobacterium* ↑ *Pseudomonas* ↓ *Escherichia-Shigella* ↓	[36]
USA; Spain	CRC/90	V1-V2	CRC/Pt	*Eikenella* ↑ *Fusobacterium* ↑ *Bulleida* ↑ *Gemella* ↑ *Parvimonas* ↑ *Campylobacter* ↑ *Streptococcus* ↑	[37]
China	CRC/19 Control/20	V3	CRC/S Control/S	*Porphyromonadaceae* ↑ *Fusobacteriaceae* ↑ *Eubacteriaceae* ↑ *Staphylococcaceae* ↑ *Campylobacteraceae* ↑	[38]
China	CRC/8	V1-V2	CRC/Pt	*Roseburia* ↑ *Microbacterium* ↓ *Anoxybacillus* ↓	[39]
USA	CRC/22 Control/13	V2-V4; V6-V9	CRC/S Control/S	*Ruminococcus* ↑ *Subdoligranulum* ↑ *Bifidobacteriaceae* ↓ *Lactobacillaceae* ↓ *Lachnoclostridium* ↓ *Oscillibacter* ↓	[40]
France	CRC/60 Control/119	V3-V4	CRC/S Control/S	All bacteria are similar in CRC and Control, respectively.	[41]
China	CRC/46 Control/56	V1-V3	CRC/S, Pt, M Control/S, M	*Lactobacillales* ↑ *Fusobacterium* ↑ *Porphyromonas* ↑ *Peptostreptococcus* ↑ *Mogibacterium* ↑ *Faecalibacterium* ↓ *Bifidobacterium* ↓ *Blautia* ↓	[42]
USA	CRC/95	V3-V5	CRC/Pt	*Fusobacterium* ↑	[43]
Brazil	CRC/15	V4	CRC/Pt	*Fusobacterium nucleatum* ↑	[44]
China	CRC/50; Control/50	V3-V4	CRC/S Control/S	*Gammaproteobacteria* ↑ *Enterobacteriaceae* ↑ *Fusobacteriales* ↑	[45]
China	CRC/52 CRA/47 Control/61	Not mention	CRC/M CRA/M Control/M	*Bacteroides fragilis* ↑ *Fusobacterium* ↑	[46]
China	CRC/8 CRA/10 Control/10	V1-V2	CRC/T CRA/T Control/T	The driver bacterial cluster is significantly and positively correlated to the pro-inflammatory passenger bacterial cluster	[47]

CRC: Colorectal cancer; CRA: Colorectal adenoma; T: Tissue; Pt: Paired tissue (CRC tissues and adjacent non-cancer tissues); S: Stool; M: Mucosa; Upward arrows indicate increase of bacterial number; Down arrows indicate decrease of bacterial number.

**Table 2:** Altered bacterial populations in CRC patients compared to healthy individuals, and in CRC tissues compared to adjacent non-cancer tissues.

Bacteria	CRC Patients vs. Healthy Individuals		CRC Tissues vs. Adjacent Non-Cancer Tissues	
	Ref. (↑in CRC)	Ref. (↓in CRC)	Ref. (↑in CRC)	Ref. (↓in CRC)
*Porphyromonas*	[32,33,35,42,45,47]	-	[30]	-
*Parabacteroides*	[31,32]	-	-	[30,37,42]
*Bacteroides*	[31]	[34,35]	[36]	[44]
*Prevotella*	[33]	[28,34]	[30,36]	[44]
*Paraprevotella*	[42]	-	-	[42,44]
*Butyricimonas*	[31,32]	-	[37]	-
*Akkermansia*	[32,34,37]	-	-	-
*Campylobacter*	[33,38,46]	-	[37,44]	-
*Desulfovibrio*	[31,42,46]	-	-	-
*Sphingomonas*	-	[31,36,46]	-	-
*Escherichia/* *Shigella*	[33,35,36,45]	[31]	-	-
*Klebsiella*	[42]	[28,31,33]	-	-
*Acinetobacter*	-	[31,36]	-	[30]
*Pseudomonas*	-	[31,36]	-	[30,36,44]
*Fusobacterium*	[32,33,36,38,42,45,46]	-	[30,36,37,43,44]	-
*Leptotrichia*	[46]	[28]	-	[28]
*Blautia*	-	[33,42,46]	-	-
*Roseburia*	[31]	[35]	[39]	-
*Lachnospira*	[31]	[42]	-	[37]
*Anaerostipes*	[31]	[33,42]	-	-
*Streptococcus*	[33,35,46,47]	-	[36]	-
*Lactococcus*	[36]	-	[36]	[30]
*Bacillus*	-	[31]	-	[30,42]
*Clostridium*	[31,32,46]	-	[44]	-
*Eubacterium*	[33,42]	[28,35]	[33]	-
*Parvimonas*	[33,45,46]	-	[30,44]	-
*Peptostreptococcus*	[32,33,35,36,42,46]	-	[30,44]	-
*Mogibacterium*	[33,42,46]	[28]	-	-
*Phascolarctobacterium*	[31,33]	[28]	-	[42]
*Oscillospira*	[31,32,46]	-	-	-
*Ruminococcus*	[31,32,33,40]	-	[44]	-
*Faecalibacterium*	-	[33,42,45,46]	-	-

**Table 3:** Basic characteristics of twelve genera of bacteria.

Taxonomy *	Genera	Characteristics	Relationship with CRC
*Bacteroidetes* *Bacteroidia* *Bacteroidales* *Bacteroidaceae*	*Bacteroides*	Gram-negative, anaerobic, non-motile, rod-shaped.	ETBF secretes BFT to promote CRC development
*Bacteroidetes* *Bacteroidia* *Bacteroidales* *Porphyromonadaceae*	*Porphyromonas*	Gram-negative, anaerobic, non-motile, rod-shaped.	*Porphyromonas* can activate inflammatory responses, may accelerate CRC development.
*Fusobacteria*; *Fusobacteriia* *Fusobacteriales* *Fusobacteriaceae*	*Fusobacterium*	Gram-negative, obligate anaerobic, non-motile, spindle-shaped.	*Fusobacterium* destroys the intestinal barrier, activates Wnt/β-catenin pathway, and promotes CRC development.
*Fusobacteria* *Fusobacteriia* *Fusobacteriales* *Leptotrichiaceae*	*Leptotrichia*	Gram-negative, anaerobic, non-motile, straight or slightly curved.	It is still unclear and needs further research.
*Verrucomicrobia* *Verrucomicrobiae* *Verrucomicrobiales* *Akkermansiaceae*	*Akkermansia*	Gram-negative, obligate anaerobic, non-motile, elliptical-shaped.	*Akkermansia* influences efficacy of PD-1-based immunotherapy against CRC
*Proteobacteria* *Epsilonproteobacteria* *Campylobacterales* *Campylobacteraceae*	*Campylobacter*	Gram-negative, aerobic or anaerobic, motile, curved-shaped.	*C. jejuni* can promote CRC development through the action of CDT
*Proteobacteria* *Deltaproteobacteria* *Desulfovibrionales* *Desulfovibrionaceae*	*Desulfovibrio*	Gram-negative, obligate anaerobic, motile, curved- or spiral rod- shaped.	*Desulfovibrio* produces hydrogen sulphide, which can promote oxidation and DNA damage to promote CRC development
*Proteobacteria* *Gammaproteobacteria* *Enterobacterales* *Enterobacteriaceae*	*Escherichia/Shigella*	Gram-negative, facultative anaerobic, non-motile, rod-shaped.	*pks*^+^*E. coli* secretes toxins, which can attack host DNA directly, increase mutation frequency and risk of CRC
*Firmicutes* *Bacilli* *Lactobacillales* *Streptococcaceae*	*Streptococcus*	Gram-positive, Facultative anaerobic/obligate, anaerobic, non-motile, round- or ovoid- shaped.	*S. gallolyticus* aggravates the tumor microenvironment thereby accelerating CRC development
*Firmicutes* *Clostridia* *Clostridiales* *Clostridiaceae*	*Clostridium*	Gram-positive, anaerobic, motile, rod-shaped.	*C. difficile* can secrete toxins, increase permeability of intestinal barrier, and promote CRC development *C. butyricum* can relieve intestinal inflammation, improve immune homeostasis, and inhibit CRC development
*Firmicutes* *Tissierellia* *Tissierellales* *Peptoniphilaceae*	*Parvimonas*	Gram-positive, obligate anaerobic, non-motile, round-shaped.	It is still unclear and needs further research.
*Firmicutes* *Clostridia* *Clostridiales* *Peptostreptococcaceae*	*Peptostreptococcus*	Gram-positive, obligate anaerobic, nonmotile, round- or oval- shaped.	*P. anaerobius* can activate NF-κB and enhance cell proliferation, subsequently accelerates CRC development

* indicating phylum, class, order, and family in different indented lines, respectively.

**Table 4:** Mechanisms for bacterial colonization in the intestine.

Bacteria	Colonization Factor	Host Colonization Target	Ref.
*B. fragilis*	*ccf*	IgA	[48,49]
	Metalloproteinase 2	E-cadherin	[50]
*F. nucleatum*	Fap2	Gal-GalNAc, overexpressed in CRC cells	[51]
	FadA	E-cadherin	[26]
*A. muciniphila*	Substrate mucin	MUC1, increased in colon cancer	[52]
		MUC5AC, absent from normal colon	
*C. jejuni*	CadF	Fibronectin	[53]
	FlpA	Fibronectin	[54]
	PEB1	-	[55]
	FliD	Heparan sulfate glycosaminoglycan receptors	[56]
*E. coli*	Intimin	-	[57]
	Afa-1	-	[58]
*S. gallolyticus*	-	Collagen I, increased in serum and tissues of CRC patients	[59,60,61]
	-	Collagen IV, highly expressed in desmoplasia of CRC liver metastases patients	
*C. difficile*	SlpA	-	[62]
	Cwp66	-	[63]
	Fbp68	Fibronectin	[64]
	CbpA	Collagens I, increased in serum and tissues of CRC patients	[64]
		Collagens V	
*P. anaerobius*	PCWBR2	α2/β1 Integrin, overexpressed in CRC tissues	[65]
